# Noninvasive Glucose Measurements in Tissue Simulating Phantoms Using a Solid-State Near-Infrared Sensor

**DOI:** 10.3390/s25072238

**Published:** 2025-04-02

**Authors:** Ariel B. Kauffman, Ruben Shakya, Shuai Yu, Mark A. Arnold

**Affiliations:** 1 Department of Engineering, Rockley Photonics, Irvine, CA 92612, USA; 2 Department of Chemistry, University of Iowa, Iowa City, IA 52242, USA

**Keywords:** noninvasive glucose monitor, noninvasive glucose sensing, near-infrared spectroscopy, solid-state laser spectroscopy, photonic integrated chips, skin glucose phantoms, RMS spectral noise benchmarking

## Abstract

Benchmark data are reported for a solid-state laser-based near-infrared spectrometer designed for noninvasive measurements in human skin. These data were obtained using a set of aqueous phantoms composed of polystyrene beads, triton X-100, saline, and glucose. The performance of this prototype solid-state laser platform was compared to parallel results obtained with a Fourier-transform (FT) spectrometer. The fundamental spectroscopic performances of the two spectrometer systems were quantified by an analysis of 100% lines determined by ratioing back-to-back spectra collected over time for each phantom. Root mean square (RMS) noise levels were computed for each dataset and the median RMS noise levels were 327.8 µAU and 667.2 µAU for the FT spectrometer and prototype laser platform, respectively. The analytical utility of the solid-state laser platform was assessed through a series of leave-one-phantom-out partial least squares analyses. Results for the laser prototype data included a standard error of cross validation (SECV) of 7.82 mg/dL for an optimized PLS model with 10 factors over a spectral range of 1401–2238 nm. This compares favorably with the results from the FT spectrometer of an SECV of 6.62 mg/dL with 8 factors and a spectral range of 1551–2378 nm. The additional two PLS factors for the laser prototype were shown to be a consequence of its higher spectral noise. Selectivity of these PLS models was demonstrated by comparing models associated with correct and random glucose assignments to each spectrum. Overall, these findings benchmark the analytical utility of this solid-state laser prototype.

## 1. Introduction

In 2021, the International Diabetes Foundation estimated that 537 million people were living with diabetes worldwide and that this number will increase to 643 million by the year 2030 [1]. Studies have shown that tight glycemic control, through frequent blood glucose monitoring, can delay the onset of diabetes-related medical complications, including retinopathy, neuropathy, and nephropathy [2,3]. Despite the benefits of tight glycemic control, the pain, complexity, and cost of contemporary home blood glucose measurements remain as key barriers to frequent glycemic testing [4,5].

The availability of a noninvasive glucose monitor (NIGM) could overcome these barriers as they relate to both invasive and minimally invasive analytical methods, such as test-strip glucose meters and continuous glucose monitors (CGMs). Although glucose meters provide high measurement accuracy across the full clinical range of blood glucose concentrations, the collection of a capillary blood sample is painful, the measurement can be messy, particularly when performed in public, and the cost per measurement can be a financial burden, especially when 6–10 measurements are recommended daily for tight glycemic control. CGM devices offer the advantages of being factory calibrated and of providing glucose concentrations continuously. These features of CGMs facilitate the documentation of valuable clinical metrics, such as time in range (TIR), time in tight range (TITR), and glycemic variability (GV). Continuous readings also make possible hybrid feedback glycemic control strategies when combined with an insulin infusion system [6,7]. Data from a wearable NIGM could also drive continuous glycemic control, but without the need to insert a fresh electrochemical biosensor subcutaneously after a week or two of operation.

A wide range of techniques have been explored as a means to measure the concentration of glucose in people noninvasively. These approaches can be split between (1) noninvasive sample collection methods and (2) noninvasive optical measurements [8]. In the first case, the concentration of glucose is measured in a sample of sweat, tear fluid, saliva or breath by using a standard analytical method, which typically must be miniaturized for this application. In the second case, electromagnetic radiation passes through a vascular region of the body and the concentration of glucose is determined from an analysis of the resulting spectrum [9,10].

Here, we are proposing the use, and further development of, near-infrared spectroscopy as a technique suitable for noninvasive glucose measurements. Near-infrared spectroscopy relies on wavelengths in the range 780–2500 nm. Over these wavelengths, absorption bands are the result of combinations and overtones of C-H, O-H, and N-H fundamental vibration modes [11]. Most biologically relevant molecules, including glucose, have absorption features over the near-infrared spectrum, thereby allowing for its potential for noninvasive glucose sensing.

It is important to recognize that the U.S. Food and Drug Administration has never approved a device for noninvasive measurements of glucose concentrations in people. Recently, Klonoff et al. put forth a call to researchers developing noninvasive glucose monitors to provide better transparency with regard to data, protocols, and results. The hope is that improved transparency will not only establish performance benchmarks and standardize criteria but will also stimulate new ideas and approaches [12].

Accordingly, this paper reports the findings of our preliminary efforts to establish the analytical utility of a solid-state laser-based phantom. In this study, near-infrared diffuse reflectance spectra are collected from a series of tissue simulating phantoms. The scattering and absorption properties of these phantoms are controlled mostly by the combination of water and polystyrene beads, respectively. These experiments represent a first step toward the development of a wearable solid-state laser-based platform for the real-time noninvasive measurement of glucose in people.

## 2. Experimental

### 2.1. Reagents

Liquid tissue phantoms were prepared by mixing solutions of Triton X-100, phosphate buffered saline, glucose, suspended polystyrene beads, and Germall Plus as a preservative. The Triton X-100, phosphate-buffered saline, glucose, and ACS reagent grade water were all sourced from the Sigma-Aldrich Chemical Company, St. Louis, MO, USA. The 10% solution of 0.308 µm polystyrene beads and Germall Plus were purchased from Bangs Laboratories, Fishers, IN, USA and Amazon, Seattle, WA, USA, respectively.

### 2.2. Tissue Simulating Phantom Construction

An array of tissue simulating phantoms was constructed as a set of forty-nine unique phantoms utilizing a 7-level Latin hypercube design [13] for which there was a total of seven unique glucose concentrations in the range 0–500 mg/dL. At each glucose concentration, a total of seven distinct phantoms were constructed across seven unique scattering levels. The polystyrene beads had an average diameter of 0.308 µm and served as the scattering bodies for this phantom set. Table 1 summarizes the seven unique concentrations used for the polystyrene beads and glucose components of the tissue phantoms. In these experiments, the concentrations of Triton X-100, saline, and Germall Plus were fixed at 800, 966, and 100 mg/dL, respectively.

Stock solutions were prepared of Triton X-100, Germall Plus, and glucose having target concentrations of 10,000 mg/dL, 2000 mg/dL, and 1500 mg/dL, respectively. The polystyrene bead mixture was used as purchased without further dilution. This mixture was composed of simply the beads suspended in water. Following preparation, all stock solutions were left to equilibrate overnight and then the density of each solution was determined by using a calibrated Blaubrand pycnometer coupled with a standard laboratory analytical balance. Density measurements were conducted in triplicate and the average value was used to determine the mass additions required for each component during phantom construction.

All liquid phantoms were prepared by using a customized Nimbus Automatic Pipetting Delivery System for the delivery of each phantom component into its sample tube. The mass addition required for each component was calculated using Equation (1):

(1) $$m_{Stock}=\frac{C_{Target}V_{Flask}}{\rho_{Stock}C_{Stock}}$$

where m_Stock_ represents the mass of each reagent to be added to the sample tube, C_Target_ is the target concentration for a given component, V_Flask_ is the volume of the volumetric flask used, ρ_stock_ is the measured density of the stock solution being dispensed, and C_Stock_ is the concentration of the stock solution. As each component was dispensed, its mass was recorded for calculation of the final component concentrations according to Equation (2):

(2) $$C_{Final}=\frac{V_{Dispensed}C_{Stock}}{V_{Sample}}$$

where C_Final_ is the final component concentration in the liquid phantom, V_Dispensed_ is the calculated volume of the stock added based on the recorded mass addition and the measured density, C_Stock_ is the calculated stock concentration, and V_sample_ is the total volume of each sample as determined as the sum of all component additions. Stock solutions were added in the following order to minimize bead aggregation: polystyrene beads, Triton-X100, water, phosphate-buffered saline, Germall Plus, and glucose. All phantom samples were sealed and stored at 5 °C until use.

### 2.3. Instrumentation and Data Acquisition

#### 2.3.1. Early-Stage Laser Prototype Spectrometer

Spectral data were collected by using an early-stage prototype of a solid-state laser spectrometer consisting of a multi-wavelength, repetitively pulsed laser array spanning both short wavelengths and combination wavelengths across the near-infrared spectrum. This spectrometer was equipped with 112 III-V semiconductor lasers housed in a monolithically integrated silicon photonic integrated chip having a peak laser power of 5 mW. For each phantom, spectral data were collected continuously for a total of seven minutes. The order of measurement for the phantoms was randomized. Spectra were collected by individually pulsing each of the 112 lasers representing a set of proprietary wavelengths with a pulse duration of 200 µs. On either side of each laser pulse sequence, a dark signal was collected for a time duration of 300 µs, which resulted in a sequence of off-times followed by the laser on-times. Lasers were pulsed in order of increasing wavelength and the resulting modulation cycle was processed to produce a single-beam spectrum.

#### 2.3.2. TTT-2500 Spectrometer

A commercially available TTT-2500 spectrometer, manufactured by TruTouch Technologies, Inc., Riverside, CA, USA, was used for comparison. The TTT-2500 is a Fourier-transform (FT)-based system developed and sold for the noninvasive spectroscopic measurement of tissue alcohol concentration. A description of the hardware has been published elsewhere [14]. The standard TTT-2500 spectrometer is equipped with a set of molded guides designed to place the subject’s fingertip or volar forearm reproducibly for a diffuse reflectance spectral measurement. For our application, the sampling guides were removed so diffuse reflectance spectra could be collected for the prepared phantoms. TTT-2500 phantom spectra were collected randomly over a period of three days.

Data acquisition parameters differed between the two systems. For data collected with the TTT-2500 instrument, the default software settings were used in which a total of eight measurements were collected in rapid succession. Each measurement represents fifteen seconds of data collection and is the result of 120 scans resulting in a total of two minutes of spectral data acquisition per phantom. Spectra were collected as double-sided interferograms having 2048 points. Interferograms were converted to single-beam spectra using a boxcar apodization with no zero-filling.

### 2.4. Temperature Control and Collection of Spectra

The same experimental procedures were followed for the collection of near-infrared spectra from both the prototype solid-state laser spectrometer and the TTT-2500 FT spectrometer. In both cases, samples were removed from the refrigerator thirty minutes prior to measurement and placed in a 32 °C temperature-controlled water bath. Following this thirty-minute thermal equilibration period, the sample vials were gently inverted 10 times prior to filtering through a 5 µm Nylon syringe filter. The filtered phantoms were then introduced into the sample chamber. A transfer pipette was used to remove any visible bubbles. The samples were allowed to equilibrate for thirty seconds with constant stirring via a magnetic stir bar in the 32 °C thermostatted sample chamber prior to initiation of spectral data collection.

**Figure 1:**
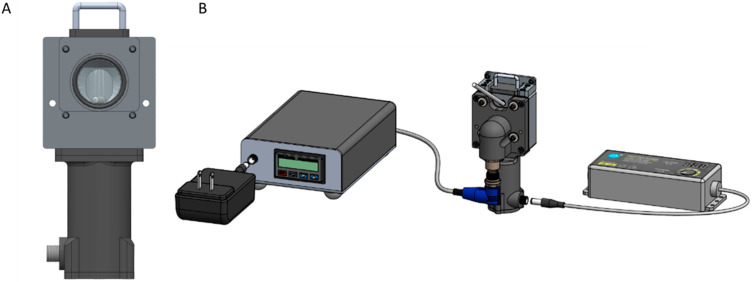
View of sample chamber highlighting (**A**) circular opening with O-ring to position the phantom sample against the optical window of the spectrometer, and (**B**) backside view showing connectivity with temperature controller and stir bar power supply.

A custom sample chamber designed to interface with both instrument systems was used for data collection. The sample chamber consisted of a cylindrical reservoir, having an internal volume of 4.5 mL, machined from stainless steel having a circular opening of 7/8 inch diameter designed to rest against the optical interface. An O-ring was positioned to seal the sample to the surface of the optical interface of each spectrometer. A 0.5-inch-long magnetic stir bar was positioned at the bottom of the sample reservoir and was rotated using a Faulhaber DC geared motor connected to an Adafruit 4880 adjustable power supply (Brooklyn, NY, USA). Temperature control of the sample chamber was achieved using a SOLO 4824 standard temperature controller, DigiKey, Thief River Falls, MN, USA. Phantom samples were measured in random order across 2–3 days of data collection. Figure 1 shows two views of the phantom sample chamber used for data collection.

A sample leak was discovered after collecting data for 1 of the 49 phantoms with the TTT-2500 spectrometer. For this reason, all data associated with this leaky phantom were discarded, and data from only 48 liquid phantoms were processed for the TTT-2500 dataset.

### 2.5. Data Transformation

Due to the inherent differences between the two instruments, the spectral data were transformed across the two data collection platforms to enable a direct comparison.

All single-beam spectra collected using the TTT-2500 instrument system were interpolated, as needed, to establish an intensity for each of the wavelengths used in the solid-state laser prototype. These interpolations used a shape-preserving piecewise cubic function resulting in a reduction in the number of *x*-axis points from 391 to 112.

Each modulation cycle for the solid-state laser prototype represents one single-beam spectrum. For each laser wavelength, the measured sample intensity was calculated as the average intensity for the laser’s on-signal minus the average dark signal, according to Equation (3). The average dark signal per laser was calculated using 100 µs of the dark signal immediately before and after the 200 µs laser pulse. Completing this process for all 112 laser wavelengths converted the modulation cycle into a 112-wavelength single-beam spectrum.

(3) $$V_{Laser}=V_{Laser\;On}-mean\left(V_{Laser\;Off\;100\mu s\;before},V_{Laer\;Off\;100\mu s\;after}\right)$$

For the raw data, the sampling rate of the TTT-2500 spectral data was 8.33 scans per second, while that for the laser prototype data was 15.15 scans per second. In order to better match these sampling rates, two consecutive intensities obtained for each wavelength from the laser system were averaged, thereby resulting in an adjusted sampling rate of 7.58 scans per second.

Similarly, the total data acquisition times were matched between the two systems. As noted above, spectral acquisition times were seven and two minutes, respectively, for the laser prototype and TTT-2500 systems. Accordingly, the first and last 2.5 min of data collected were removed for each early-stage prototype spectrum, resulting in the use of a two-minute segment of intensity data collected with the prototype spectrometer.

All data processing and analyses were performed using MATLAB 2022b (MathWorks, Natick, MA, USA).

## 3. Results and Discussion

### 3.1. Comparison of the Spectral Datasets

The raw spectra collected for each dataset were pre-processed to provide comparable spectra. As described above, these pre-processing steps resulted in all spectra within each of the two datasets consisting of 112 spectral resolution elements (wavelengths) collected with an approximate sampling rate of 8 scans per second coupled with a two-minute effective acquisition time.

Despite being collected from different types of instruments, the relative analytical utility of these datasets can be ascertained through a comparison of the root-mean-square (RMS) noise on 100% lines. Such an analysis provides a measure of the analytical signal-to-noise ratio that incorporates spectral variations that originate from the instrumentation, the spectrometer-to-sample interface, and the ambient environment. As such, this RMS noise analysis provides a meaningful analytical benchmark of each dataset.

A 100% line is calculated as the negative base-10 logarithm of the ratio of two back-to-back single-beam spectra collected under identical experimental parameters and measurement conditions. Ideally, 100% lines present as a noise-free horizontal line crossing the *y*-axis at zero absorbance. In practice, however, 100% lines can be offset from an absorbance of zero, they can demonstrate curvature, and they always include noise.

Such non-ideal features of the 100% lines can be a diagnostic for analytical purposes in that they are derived from the differences in the sample, the instrument, or the sample-to-instrument interface between when the ratioed spectra were collected. For example, 100% lines determined from near-infrared spectra collected from aqueous solutions typically show a curvature that represents small, but significant, temperature-induced shifts in the underlying water absorption bands [15].

In this work, a set of 100% lines were computed for each phantom by ratioing sequential spectra collected with the different spectrometers. The RMS noise was calculated by fitting each 100% line to a second-order polynomial and subtracting the fitted value from the measured value. This second-order polynomial accounts for offsets, and curvatures in these 100% lines, predominantly caused by differences in scattering and temperature, respectively, for the two ratioed spectra. Offsets can also be caused by relatively moderate-term fluctuations in intensity of the incident radiation associated with diffuse reflectance measurements. The presence of non-random noise or broad spectral features in the processed 100% lines suggests changes in the sample composition, interface movement or instrument instability.

The RMS noise of the processed 100% lines can be calculated from Equation (4):

(4) $$RMS\;Noise=\sqrt{\frac{\sum_{i=1}^{n}{\left(y_{i}\right)}^{2}}{N-1-k}}$$

where *y* represents the magnitude of the corrected 100% line across a chosen wavelength region, *N* represents the number of wavelengths over the specified region and *k* represents the polynomial order used for fitting of the 100% lines. The RMS noise is provided in units of micro-absorbance (µAU). The signal-to-noise ratio (SNR) of the analytical measurement can be obtained from the RMS noise level according to Equation (5).

(5) $$SNR={\left(2.303\times RMS\;Noise\right)}^{-1}$$

**Figure 2:**
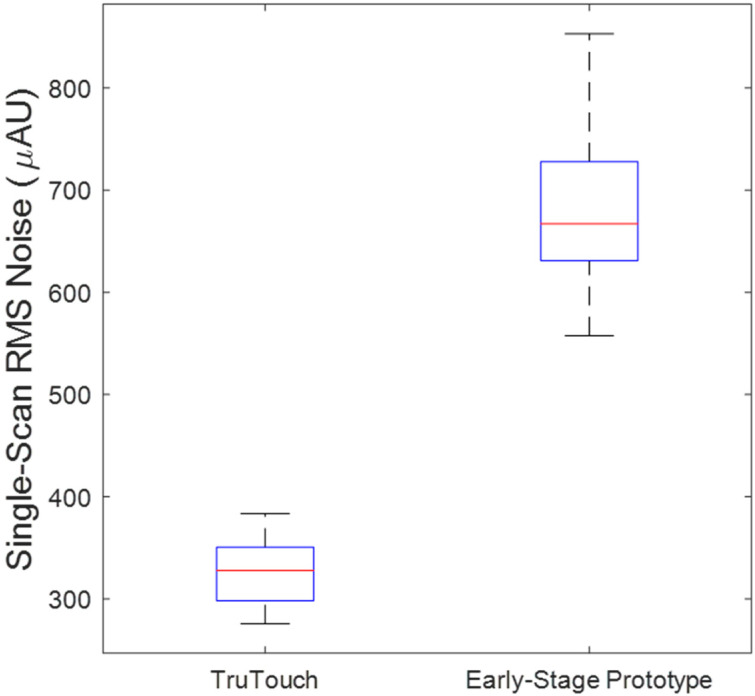
Comparison of single-scan RMS noise values for TTT-2500 and early-stage prototype instruments.

Accordingly, RMS noise values (in units of micro-absorbance, µAU) were determined from all spectra collected across each of the phantoms for the two instrument systems. The results are summarized in the boxplots presented in Figure 2. Median RMS noise values for the TTT-2500 and laser prototype spectrometer phantom measurements were 327.8 µAU and 667.2 µAU, respectively. Accordingly, these RMS values, and the corresponding SNRs, indicate the performance of the prototype laser system is about half of that for the FT spectrometer. These RMS levels correspond to single-scan spectra collected over 0.12 second and 0.13 second, respectively, for these two spectrometer systems.

The above RMS noise levels can be normalized for a one-second scan as follows. Assuming the presence of white noise and theoretical signal averaging as a function of $\frac{1}{\sqrt[{2}]{N}}$ where *N* represents the number of averaged spectra, the theoretical RMS noise of each instrument system can be calculated for a one-minute measurement. The median theoretical RMS noise values for a one-minute spectra measurement are 15.0 µAU and 33.8 µAU for the TTT-2500 and laser spectrometers, respectively. Using Equation (5), these theoretical RMS noise values for one-minute measurements correspond to SNR values of 28,900 and 12,800 for the TTT-2500 and the laser spectrometers, respectively.

### 3.2. Partial Least Squares Analysis

For each dataset, separate PLS calibration models were generated for quantitation of glucose in the phantoms. The diffuse reflectance spectra were transformed as the negative base-10 logarithm of the pre-processed single-beam spectra. The transformed spectra were averaged per each phantom resulting in a single two-minute spectrum for each phantom. In total, datasets consisted of 48 and 49 transformed two-minute spectra for the TTT-2500 and early-stage prototype spectrometer systems, respectively.

PLS calibration models were generated by using a leave-one-phantom-out approach. This method provides an estimate of analytical performance in situations where the number of spectral data is limited, as is the case here with ≈49 spectra across seven different glucose concentrations and seven unique scattering matrices (i.e., levels of polystyrene beads). The calculation involved creating a PLS model when the spectrum of one phantom was left out and then predicting the concentration of glucose for this left-out spectrum. The standard error of cross validation (SECV) is a measure of the accuracy of the glucose concentration predictions summed for each of the left-out phantom spectra. The SECV can be calculated from Equation (6) where $y_{i}$ and $\hat{y_{i}}$ represent the actual and predicted concentrations, respectively, and N represents the total number of data points used in the calculation of the SECV. The optimum model corresponds to the lowest value for the standard error of cross-validation (SECV).

(6) $$SECV=\sqrt{\frac{\sum_{i=1}^{n}{\left(y_{i}-\;\hat{y_{i}}\right)}^{2}}{N}}$$

Both spectral range and the number of latent variables, or factors, were examined to optimize performance of the PLS calibration model. A grid search algorithm was used to determine the optimal wavelength range for each dataset. This algorithm was initiated with a minimum window size of 50 nm and this window size was increased incrementally by 50 nm until the window size matched the fully collected spectral range. For each spectral range, SECV values were recorded for different PLS models using 1 to 20 latent variables.

Table 2 provides a comparison of the optimum PLS models generated for each spectrometer type. The grid search algorithm resulted in similar wavelength ranges being used across the two instruments. Both models utilized the entirety of the first overtone region (1540–1820 nm) and the majority of the available combination region wavelengths (2000–2500 nm). The TTT-2500 model utilized eight factors compared to the ten factors found to be optimal for the prototype instrument. This increase in the number of latent variables for the laser prototype system is consistent with the higher noise levels associated with these spectra.

**Figure 3:**
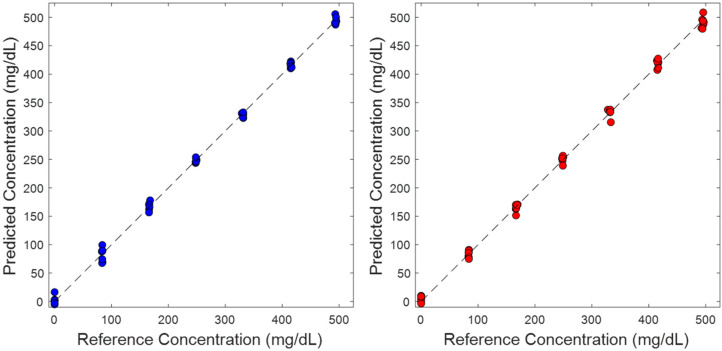
Concentration correlation plots for (**left**) TTT-2500 and (**right**) early-stage prototype instruments in which the dashed black line represents the unity line and circles represent the measured leave-one-phantom out cross-validation values.

Figure 3 provides a visual comparison of results in the form of two concentration correlation plots, one for the TTT-2500 (blue circles) and one for the laser prototype (red circles). In both cases, the concentrations of glucose predicted from the corresponding PLS model are compared to the reference value for each phantom. The unity line is provided to represent the ideal result where the predicted concentrations equal the reference concentrations.

For both PLS models (TTT-2500 model and laser-prototype model), all concentration predictions lie along the unity line indicating excellent agreement between the reference and predicted data. Likewise, Table 2 provides the results of a linear regression analysis of each dataset and, in both cases, the slope and y-intercept match the unity line. For each glucose concentration, the tight cluster of data points represents the seven distinct phantoms with each having unique scattering properties as a result of varying concentrations of polystyrene beads.

### 3.3. Impact of Additional Random Noise

To better understand the increased number of factors required for the early-stage prototype PLS model, random noise was added to the TTT-2500 spectral data such that the calculated RMS noise was similar to that determined for the early-stage prototype spectra. The calculated RMS noise on the TTT-2500 plus random noise spectra had a median RMS value of 673.3 µAU, which is similar to the median RMS noise value of 667.2 µAU (See Figure 2) for the early-stage prototype.

Implementing the same data analysis method described above, an analysis of the modified TTT-2500 dataset resulted in the same spectral range as listed in Table 2 for the laser prototype data. In addition, the resulting PLS model for the modified TTT-2500 dataset had a SECV equal to 8.81 mg/dL and utilized a total of ten factors. The increase in SECV and the need for two additional PLS factors suggests that the additional two factors required for the laser prototype spectral data are compensating for the higher RMS noise levels associated with the laser prototype.

This analysis is unable to assess the major sources of the elevated random noise observed in the laser-prototype data. Possibilities include instabilities of the spectrometer components, variations in the positioning of the interface between the spectrometer and the phantom, and uncontrolled changes in the ambient environment. Each of these potential sources of spectral variances can cause both systematic (non-random) and non-systematic (random) variances that can contribute to measurement errors and reduced analytical performance, such as limit of detection. The PLS algorithm can account for non-random spectral variances if they are properly represented in the training set. If not, then such variances can adversely impact measurement accuracy and overall analytical performance.

### 3.4. Randomized Glucose Concentration Assignments

**Figure 4:**
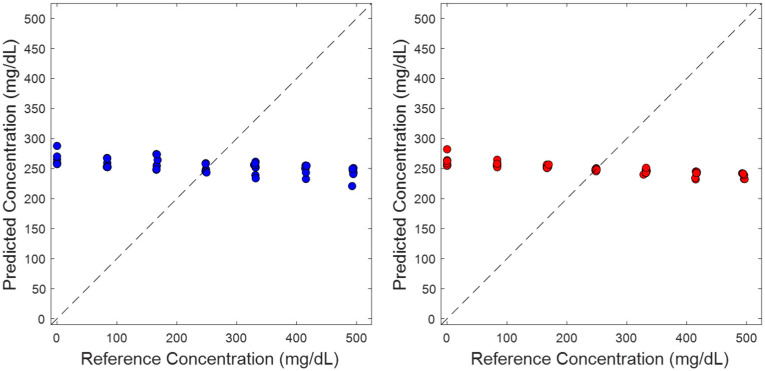
Concentration correlation plots for (**left**) TTT-2500 and (**right**) early-stage prototype instruments in which glucose concentration assignments were randomized prior to generation of a leave-one-phantom-out cross-validation PLS model. The dashed black line represents the unity line and circles represent predicted concentrations.

In an effort to demonstrate that the PLS calibration models were derived from glucose-specific spectral information, glucose concentrations were randomized with respect to their originally assigned values for each spectrum. Leave-one-phantom-out cross-validation models were generated having the same spectral ranges presented in Figure 4. The resulting PLS models had SECV values of 169.87 mg/dL and 173.32 mg/dL for TTT-2500 and early-stage prototype instrument systems, respectively, with no correlation between reference and predicted glucose concentrations. The standard deviation of the glucose concentrations across the phantom datasets was approximately 167 mg/dL, which is similar in magnitude to the SECV values achieved with random glucose assignment. The similarity of SECV and glucose concentration standard deviation indicates that these PLS models were predicting the average glucose concentration. This finding is a direct result of the bias-variance trade-off and signifies the inability of the PLS model to find spectral information that correlates with the analyte of interest [16].

**Figure 5:**
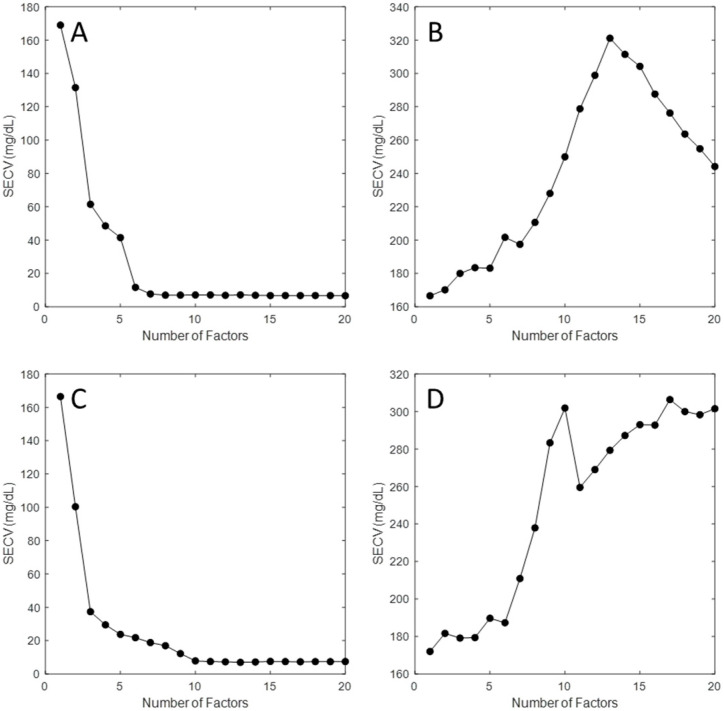
Standard error of cross-validation as a function of the number of PLS factors for the TTT-2500 (**A**,**B**) and early-stage laser prototype (**C**,**D**) spectrometers for PLS calibration models when using correct (**A**,**C**) and random (**B**,**D**) glucose concentration assignments.

In addition to significant differences in prediction performance, differences are visible for plots of SECV as a function of the number of factors used in the PLS model, as shown in Figure 5 for both TTT-2500 and early-stage prototype systems. Ideally, increasing the number factors would result in a drop in SECV, as shown for the PLS models using the correctly assigned glucose concentrations. When glucose concentrations are assigned randomly, the SECV increases with an increasing number of factors, which provides further evidence in the algorithm’s inability to extract a glucose-selective signal.

## 4. Conclusions

This work demonstrates the analytical utility of this prototype laser-based solid-state spectrometer for the quantification of glucose in a scattering, aqueous matrix. Similar analytical performance was demonstrated between spectra collected with the novel laser spectrometer system in direct comparison to a research-grade FT spectrometer. SECV values were 7.82 and 6.62 mg/dL for these two datasets, respectively. These similar SECV values required a PLS model with two additional latent variables for the laser prototype to accommodate higher noise levels. Our analysis illustrates that the SNR for the prototype laser system was only half that of the FT spectrometer, thereby providing a tangible benchmark for future design improvements. Specificity for glucose was illustrated by characterizing PLS models with randomly, incorrect glucose concentration assignments.

The in vitro results presented here represent a first step in the development of a solid-state laser system for noninvasive glucose sensing. Although the simplicity of the phantom samples is appropriate for this initial feasibility study, it must be recognized that measurements in living skin will be considerably more complicated. In these phantoms, variations in the diffuse reflectance near-infrared spectra are caused by changes in the concentrations of both glucose and polystyrene beads, as well as the amount of water in the optical path associated with water displacement effects. For living skin, diffuse reflectance spectra will be heavily impacted by variations in both the scattering and absorption properties associated with a myriad of skin components.

Comparison of the proposed laser prototype to a FT spectrometer is motivated by the difficulty in comparing analytical performance across near infrared systems reported by others. FT spectrometers are widely recognized for their spectral stability, generally attributed to high radiant throughput and superb signal averaging capabilities. These features are well suited as a high-level comparator for development of the proposed laser prototype. Comparisons to results reported by others are complicated by several factors, including (1) a lack of defined conditions, (2) a lack of agreed upon spectral metrics, and (3) a lack of benchmark values. In general, the *in vivo* detection of glucose in people with diabetes requires a limit of quantitation suitable for detecting hypoglycemia (50–70 mg/dL) [17,18]. The SECV values reported here are well under this physiological threshold, but it must be recognized that living skin is more complex and variable than the static phantoms used here.

Overall, this work establishes the analytical utility of the proposed solid-state system, thereby justifying its further development, refinement, and testing in more complex matrixes.

## Figures and Tables

**Table 1:** Concentrations of glucose and scatterers in tissue phantoms *.

Glucose	0	82.5	167.5	250	332.5	417.5	500
Polystyrene beads	1466	1588	1712	1734	1956	2078	2200

* Concentrations in units of mg/dL.

**Table 2:** Optimized PLS parameters and results for TTT-2500 and prototype instrument systems.

Instrument	TTT-2500	Early-Stage Prototype
Spectral range (nm)	1551–2378	1401–2238
Number of factors	8	10
SECV (mg/dL)	6.62	7.82
Regression slope	0.998 (±0.006)	0.998 (±0.007)
Regression intercept	1.0 (±2)	1.0 (±2)
Coefficient of determination, R^2^	0.998	0.998

## Data Availability

The datasets presented in this article are not available due to commercial restrictions.
